# Effect of decanoic acid and 10-hydroxydecanoic acid on the biotransformation of methyl decanoate to sebacic acid

**DOI:** 10.1186/s13568-018-0605-4

**Published:** 2018-05-05

**Authors:** Yohanes Eko Chandra Sugiharto, Heeseok Lee, Annur Dyah Fitriana, Hyeokwon Lee, Wooyoung Jeon, Kyungmoon Park, Jungoh Ahn, Hongweon Lee

**Affiliations:** 1 Biotechnology Process Engineering Center, Korean Research Institute of Bioscience and Biotechnology (KRIBB), 30 Yeongudanji-ro, Cheongwon-gu, Cheongju-si, Chungcheongbuk-do 28116 Republic of Korea; 2 Department of Bioprocess Engineering, KRIBB School of Biotechnology, Korea University of Science and Technology (UST), 217 Gajeong-ro, Yuseong-gu, Daejeon, 34113 Republic of Korea; 3 Department of Biological and Chemical Engineering, Hongik University, 2639 Sejong-ro, Sejong-si, 30016 Republic of Korea

**Keywords:** Sebacic acid, Methyl decanoate, ω-oxidation, 10-hydroxydecanoic acid, Decanoic acid, Decane induction

## Abstract

Biotransformation of fatty acid methyl esters to dicarboxylic acids has attracted much attention in recent years; however, reports of sebacic acid production using such biotransformation remain few. The toxicity of decanoic acid is the main challenge for this process. Decane induction has been reported to be essential to activate the enzymes involved in the α,ω-oxidation pathway before initiating the biotransformation of methyl decanoate to sebacic acid. However, we observed the accumulation of intermediates (decanoic acid and 10-hydroxydecanoic acid) during the induction period. In this study, we examined the effects of these intermediates on the biotransformation process. The presence of decanoic acid, even at a low concentration (0.2 g/L), inhibited the transformation of 10-hydroxydecanoic acid to sebacic acid. Moreover, about 24–32% reduction in the decanoic acid oxidation was observed in the presence of 0.5–1.5 g/L 10-hydroxydecanoic acid. To eliminate these inhibitory effects, we applied substrate-limiting conditions during the decane induction process, which eliminated the accumulation of decanoic acid. Although the productivity of sebacic acid (34.5 ± 1.10 g/L) was improved, by 28% over that achieved using the previously methods, after 54 h, the accumulation of 10-hydroxydecanoic acid was still detected. The accumulation of 10-hydroxydecanoic acid even under the decane limiting conditions could be an evidence that oxidation of 10-hydroxydecanoic acid could be the rate-limiting step in this process. The improvement of this reaction should be an important objective for further development of the production of sebacic acid using biotransformation.

**Electronic supplementary material:**

The online version of this article (10.1186/s13568-018-0605-4) contains supplementary material, which is available to authorized users.

## Introduction

Sebacic acid, a 10 carbon containing dicarboxylic acid (DCA), is an important precursor in the production of nylon and polyamides (PAs), primarily PA-4,10 and PA-5,10 (Chung et al. 2015). Like other medium-chain DCAs, the commercial process for the production of sebacic acid depends on chemical methods, principally involving alkaline oxidation of vegetable oils, such as castor oil, in which 2-octanol is generated as a byproduct (Green et al. 2000; Azcan and Demirel 2008; Metzger 2009). However, the production of medium-chain DCAs through chemical routes has several problems, particularly the use of harsh production conditions and generation of by-products. Biological processes for the production of DCAs can overcome these limitations because they require milder conditions (Huf et al. 2011; Song et al. 2013).

Recently, the biological production of DCAs, especially via diterminal oxidation by hydrocarbon-degrading microorganisms or metabolic engineering of other microorganisms, such as *E*. *coli*, has been a focus of several studies (Yi and Rehm 1982; Picataggio et al. 1992; Liu et al. 2004; Sathesh-Prabu and Lee 2015; Cao et al. 2017; Lee et al. 2017; Yu et al. 2017). One of the commonly reported pathways for the production of DCAs involves diterminal oxidation of alkanes, resulting in the conversion of both the terminal methyl groups into carboxyl groups. The entire conversion process is referred to as the α,ω-oxidation pathway (Kester and Foster 1963; Ratledge 1984). The mechanisms of oxidation in this pathway have been extensively studied (Eschenfeldt et al. 2003; Cheng et al. 2005; Huf et al. 2011; Werner and Zibek 2017). Remarkably, this biotransformation process appears to be more feasible today for producing DCAs, and metabolic engineering of *E. coli* could be a breakthrough technology for production of these compounds either from glucose or fatty acids in the future.

Despite the popularity of alkanes as the main substrates for biotransformation processes, fatty acids and fatty acid methyl esters (FAMEs) have received more attention recently because they are considered as renewable resources. Fatty acids are naturally produced by plants whereas FAMEs are produced from transesterification of vegetable oils (Fukuda et al. 2001; Berchmans and Hirata 2008). Therefore, their use as the main raw material for DCA production can reduce the consumption of fossil fuels and would, thereby, prevent the aggravation of global warming. Recently, some researchers have elucidated the process of biotransformation of methyl laurate to dodecanedioic acid (Funk et al. 2017; Lee et al. 2017). These findings showed that *Candida tropicalis* could secrete several kinds of lipases and esterases for hydrolyzing FAMEs to fatty acids before converting them to DCAs (Galán-Ladero et al. 2010). Accordingly, FAMEs could be used as chief substrates for large-scale production of DCAs.

The biotransformation of FAMEs has been widely used for the production of dodecanedioic acid (12 carbon chain of DCAs) from dodecane (Yi and Rehm 1982; Picataggio et al. 1992), methyl dodecanoate (Funk et al. 2017), or a combination of the two (Lee et al. 2017). However, the use of this strategy for the production of sebacic acid has not been much reported (Shiio and Uchio 1971; Beardslee et al. 2014; Chung et al. 2015). Researchers have used decane induction for biotransformation of methyl decanoate to sebacic acid (Beardslee et al. 2014). Particularly, decane induction was performed using 10 g/L decane for 6 h before methyl decanoate was fed into the fermenter for biotransformation. However, while examining this method, we found the accumulation of decanoic acid and 10-hydroxydecanoic acid during the decane induction period even at lower concentrations of decane, which possibly could decrease the productivity of sebacic acid.

The toxicity of decanoic acid is considered to be a major limitation in the biological production of sebacic acid. Notably, medium-chain fatty acids, including decanoic acid, were reported to be cytotoxic (Viegas et al. 1989; Stevens and Hofmeyr 1993; Green et al. 2000; Liu et al. 2013); these compounds can either kill microorganisms or inhibit their growth (Desbois and Smith 2010). Moreover, the minimum inhibitory concentration of decanoic acid is very low. For instance, the growth of *Saccharomyces cerevisiae* was inhibited by 0.25 mM decanoic acid (Stratford and Anslow 1996) and even 12 μM decanoic acid exhibited 17% inhibition of growth in *S. cerevisiae* (Alexandre et al. 1996). Thus, the accumulation of decanoic acid inactivates the cells and ceases the production of sebacic acid. Furthermore, decanoic acid remaining in the fermenter broth could cause foaming. Similar to the production of DCAs, the presence of decanoic acid in the fermenter can decrease the pH, and alkaline solution added to restore the pH in the fermenter (Hill et al. 1986) can react with the remaining fatty acid compounds to produce soap. This phenomenon is undesirable because it drastically increases the level of broth, even to the point of overflowing from the fermenter. It is, therefore, critical to maintain decanoic acid-free conditions during the biological production of sebacic acid.

In this study, we investigated the effect of decanoic acid and 10-hydroxydecanoic acid on the α,ω-oxidation pathway. To enhance the biological production of sebacic acid, we attempted to remove their accumulation by performing decane induction using the continuous feeding method. In this process, the feeding rate of decane was regulated such that no decane remained in the fermenter broth. Thus, we used a substrate limiting condition during the induction period for sebacic acid production.

## Materials and methods

### Microorganism

A mutant of *C. tropicalis* American Type Culture Collection (ATCC) 20962 that was isolated via direct evolution using a continuous culture was used in this study. This strain has higher tolerance to decanoic acid than *C. tropicalis* ATCC 20962.

### Flask experiments

Several colonies from agar plates were transferred to 20 mL yeast mold (YM) containing 3 g/L yeast extract, 3 g/L malt extract, 5 g/L peptone, and 10 g/L dextrose in a 250-mL baffled flask and cultivated overnight. Two milliliters of the cultivated broth was transferred to the growth medium containing 75 g/L glycerol, 6.7 g/L yeast nitro base without amino acids, 10 g/L yeast extract, 3 g/L ammonium sulfate, 5.9 g/L potassium dihydrogen phosphate, and 1.15 g/L dipotassium hydrogen phosphate. In the flask experiments, glycerol was selected as the carbon source instead of glucose. The use of glucose produces many organic acids, which are unfavorable for this process. After 24 h of growth, the entire broth from the growth flask was transferred to a conversion flask. After transferring the broth, the composition of components in the conversion flask was as follows: 50 g/L glycerol, 6.5 g/L yeast nitro base without amino acids, 3 g/L yeast extract, 1.82 g/L potassium dihydrogen phosphate, and 15 g/L dipotassium hydrogen phosphate, with additional substrates (decane, decanoic acid, methyl decanoate, 10-hydroxydecanoic acid, or a mixture of these substrates). Decanoic acid was stocked as potassium decanoate, which was produced by mixing potassium hydroxide and decanoic acid in demineralized water. The flask experiments were conducted in duplicate to ensure the credibility of results.

### Fermentation experiments

Several colonies from agar plates were transferred to 20 mL YM solution and cultivated overnight. The culture was then transferred to 180 mL seed culture medium in a 2-L baffled flask and inoculated overnight. The composition of the seed culture medium was as follows: 20 g/L glucose, 5 g/L yeast extract, 0.5 g/L sodium chloride, 3.5 g/L potassium nitrate, and 5 g/L potassium dihydrogen phosphate. This culture was used as the seed for the main fermenter. The composition of the main fermenter broth was as follows: 50 g/L glucose, 15 g/L yeast extract, 0.5 g/L sodium chloride, 3.5 g/L potassium nitrate, 5 g/L potassium dihydrogen phosphate, and 0.5 g/L antifoam. The fermenter (CNS, Daejeon, South Korea) was operated at 30 °C and 1 vvm of aeration. The agitation was in the range of 600–1000 rpm. The pH of the broth was maintained at 5.5 by adding 6.5 N sodium hydroxide. After glucose depletion, glucose solution (mixture of 800 g/L glucose and 40 g/L yeast extract) was fed to the fermenter until the cell concentration reached an optical density of 80. The glucose feeding speed was between 7.5 and 12.5 g/L/h. After the desired optical density was reached, the glucose feeding rate and pH were adjusted to 2–2.5 g/L/h and 7.5, respectively. Thereafter, decane induction was conducted with either a single bolus addition/pouring (Beardslee et al. 2014), which is called the previous induction method, or the continuous feeding method. In the previous induction method, 10% v/v decane was poured into the fermenter after the growth phase was over, whereas 0.65 g/L/h decane was fed in the continuous feeding method. After completion of the induction, methyl decanoate was fed into the fermenter at a feeding rate between 0.6 and 0.7 g/L/h. When decane induction was performed by the pouring method, the methyl decanoate feeding rate was adjusted to prevent the sudden accumulation of fatty acid. The fermentation experiments were conducted in duplicate with at least three data measurements to ensure the credibility of results.

### Analytical method

The biomass concentration was analyzed by measuring the absorbance at 600 nm using a UV spectrophotometer (Uvikon XL, Secomam, France). The glucose concentration was analyzed with a glucose analyzer (YSI 2700 Biochemistry Analyzer; Yellow Springs Instrument, USA).

The concentrations of sebacic acid, decane, decanoic acid, and 10-hydroxydecanoic acid were determined using gas chromatography as explained previously (Mishra et al. 2016). Gas chromatography (DANI Master GC; DANI Instruments SpA, Italy) was performed using an Rtx-5 column (Restek Corporation, USA). The oven temperature ranged from 70 to 237 °C, and the injector and detector temperatures were 280 and 300 °C, respectively. Prior to the analysis, 100 μL culture broth was mixed with 100 μL internal standard and was acidified with 100 μL of 5 N H_2_SO_4_. Tetradecanedioic acid (10 g/L) was used as the internal standard. The acidified samples were extracted with 300–500 μL diethyl ether. The solvent phase was then separated and mixed with *N*,*O*-bis(trimethylsilyl) trifluoroacetamide/BSTFA at 2:1 (v/v). The final solution was then analyzed by gas chromatography.

## Results

### Biotransformation of methyl decanoate to sebacic acid using decane induction: current limitations

**Fig. 1:**
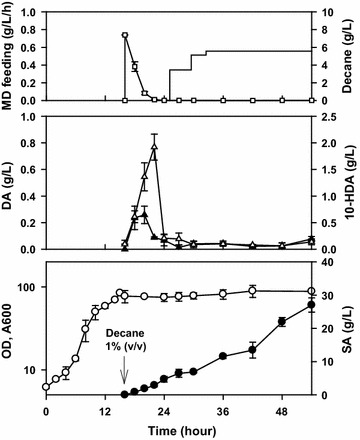
Biotransformation of methyl decanoate to sebacic acid using an evolved *Candida tropicalis* strain with decane induction method. This experiment was adapted from that described in a previous report after several modifications (Beardslee et al. 2014). After the completion of the growth phase, decane induction was conducted for 10 h using 10% v/v decane. Thereafter, methyl decanoate was fed slowly (and subsequently increased in a sequential manner) after decane induction to avoid the accumulation of decanoic acid. Error bars represent standard deviation of three measurements from two independent runs. *OD* optical density, *SA* sebacic acid, *DA* decanoic acid, *10-HDA* 10-hydroxydecanoic acid, *MD* methyl decanoate. Symbols: : optical density; : sebacic acid concentration; : 10-hydroxydecanoic acid concentration; : decanoic acid concentration; : decane concentration; : methyl decanoate feeding rate; *methyl decanoate feeding rate was counted on the initial volume basis

The biotransformation of methyl decanoate to sebacic acid was conducted in a 5-L fermenter (Fig. 1). After decane induction, methyl decanoate was fed into the fermenter, initially at a slow feeding rate (0.35 g/L/h) that was increased step-wise until it reached the desired value. The purpose of slower substrate feeding rate was to allow the cells to adapt to the toxicity of decanoic acid, which was produced from methyl decanoate. After 54 h, 27.0 ± 2.14 g/L sebacic acid (0.50 ± 0.04 g/L/h) was produced using this method.

The intermediates of the α,ω-oxidation pathway, primarily decanoic acid and 10-hydroxydecanoic acid, were observed to accumulate during the decane induction period to specific concentrations. The accumulation of decanoic acid occurred in a shorter time compared to that required for the accumulation of 10-hydroxydecanoic acid. Moreover, the concentration of 10-hydroxydecanoic acid during the accumulation period was higher than that of decanoic acid.

### Effect of decanoic acid and 10-hydroxydecanoic on the α,ω-oxidation pathway

**Fig. 2:**
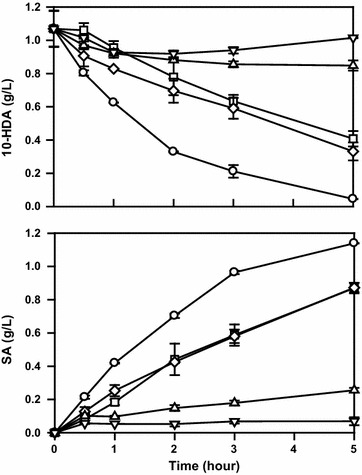
Effects of decanoic acid concentration on the production of sebacic acid from 10-hydroxydecanoic acid. Error bars represent standard deviation of two independent runs. Substrates: : 10-HDA (1 g/L); : 10-HDA (1 g/L) and DA (0.2 g/L); : 10-HDA (1 g/L) and DA (0.4 g/L); : 10-HDA (1 g/L) and DA (0.6 g/L); : 10-HDA (1 g/L) and DA (0.8 g/L)

We determined the effect of decanoic acid and 10-hydroxydecanoic acid, especially of the latter compound, accumulated during the induction period, on the α,ω-oxidation pathway because very little information is available in this regard, as of date. Firstly, the toxicity of decanoic acid was tested in the flask culture during the production of sebacic acid from 10-hydroxydecanoic acid (Fig. 2). In the absence of decanoic acid, *C. tropicalis* completely converted 1 g/L of 10-hydroxydecanoic acid to sebacic acid after 5 h in the flask culture. However, decanoic acid, even at 0.2 g/L, inhibited the conversion of 10-hydroxydecanoic acid to sebacic acid. Furthermore, there was almost no 10-hydroxydecanoic acid conversion when the decanoic acid concentration was higher than 0.4 g/L. These results indicated that decanoic acid inactivated the cells when its concentration exceeded the inhibitory concentration for this strain (0.4 g/L).

**Fig. 3:**
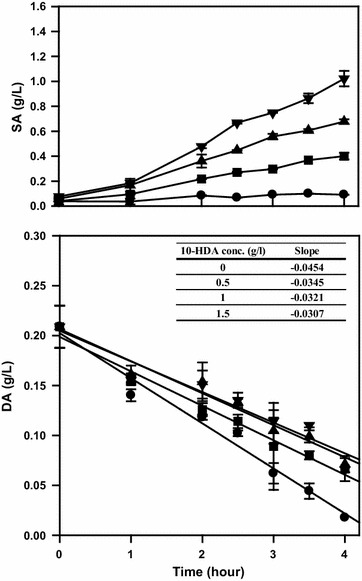
Effects of 10-hydroxydecanoic acid concentration on decanoic acid oxidation. Error bars represent standard deviation of two independent runs. Substrates: : DA (0.2 g/L); : DA (0.2 g/L) and 10-HDA (0.5 g/L); : DA (0.2 g/L) and 10-HDA (1 g/L); : DA (0.2 g/L) and 10-HDA (1.5 g/L)

Next, the effects of 10-hydroxydecanoic acid on the rate of decanoic acid oxidation were examined in the flask culture (Fig. 3). The consumption rate of decanoic acid in the presence of 10-hydroxydecanoic acid (between 0.5 and 1.5 g/L) was 24–32% lower than in the absence of 10-hydroxydecanoic acid, showing the inhibition of the decanoic acid oxidation reaction. Additionally, the amount of sebacic acid produced was proportional to the initial concentration of 10-hydroxydecanoic acid; sebacic acid was mainly produced from 10-hydroxydecanoic acid in this experiment.

**Fig. 4:**
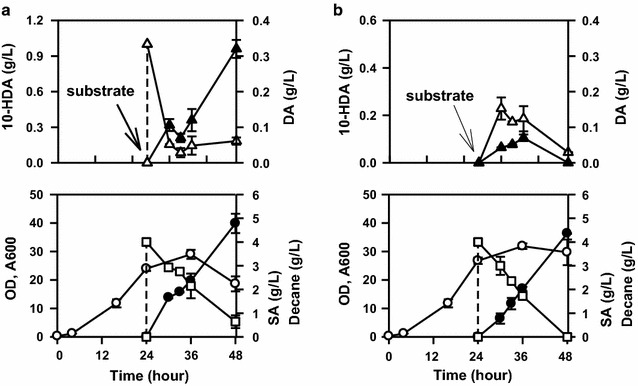
Effects of 10-hydroxydecanoic acid on the oxidation of alkanes. The substrates were as follows: **a** 4 g/L decane and 1 g/L 10-HDA; **b** 4 g/L decane. Error bars represent standard deviation of two independent runs. Symbols: : optical density, A600; : sebacic acid concentration; : decane concentration; : 10-hydroxydecanoic acid concentration; : decanoic acid concentration

Subsequently, the effect of 10-hydroxydecanoic acid on the oxidation of decane was examined in a flask culture (Fig. 4a). The presence of 10-hydroxydecanedioic acid triggered the accumulation of decanoic acid (from the oxidation of decane) and cell deactivation after 12 h of induction (36 h of cultivation). This phenomenon did not occur when there was no 10-hydroxydecanoic acid in the broth (Fig. 4b). In addition, the decane consumption rate was decreased when 10-hydroxydecanoic acid was present in the broth.

Based on these data, we concluded that both the intermediates negatively affect the biological production of sebacic acid. Therefore, their elimination is important for the robustness of the process.

### Decane induction by continuous feeding method

**Fig. 5:**
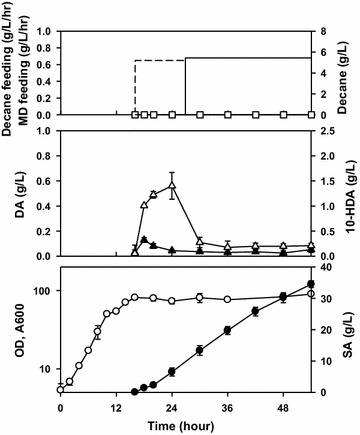
Biotransformation of decane and methyl decanoate to sebacic acid using an evolved *Candida tropicalis* strain. In this experiment, decane was fed continuously during the induction period before the transformation of methyl decanoate. Error bars represent standard deviation of four measurements from two independent runs. Symbols: : optical density; : sebacic acid concentration; : 10-hydroxydecanoic acid concentration; : decanoic acid concentration; : decane concentration; : methyl decanoate feeding rate; : decane feeding rate; *decane and methyl decanoate feeding rates were counted based on the initial volume basis

To prevent the accumulation of decanoic acid and 10-hydroxydecanoic acid, a modification of decane induction method, by continuous feeding instead of pulse pouring, was performed in a 5-L fermenter. After completion of the growth phase, approximately 0.65 g/L/h decane was fed into the fermenter for induction (Fig. 5). Importantly, decane feeding rate was adjusted such that there was no decane remaining in the fermenter broth. After approximately 10 h of induction, methyl decanoate was fed at a similar feeding rate. Approximately 34.5 ± 1.10 g/L sebacic acid (0.64 ± 0.02 g/L/h) was produced after 54 h of fermentation using this method. In this method, the fermenter broth contained almost no decanoic acid. However, the accumulation of 10-hydroxydecanoic acid in the fermenter broth was still observed.

## Discussion

As observed in previous studies, decane induction was necessary for the activation of enzymes involved in the α,ω-oxidation pathway prior to the biotransformation of methyl decanoate to sebacic acid (Beardslee et al. 2014). In a preliminary test (Additional file 1: Figure S1), the possibility of using potassium decanoate (represented as decanoic acid) or methyl decanoate as inducers for these enzymes was examined in flask cultures. The results of this experiment confirmed that the toxicity of decanoic acid interferes with the activation and even inactivates the cells (when potassium decanoate or methyl decanoate were used as inducers at concentrations as low as 0.4 g/L). In contrast, this negative trend was not observed when decane was used for induction. Therefore, decane induction was inevitable for the biotransformation of methyl decanoate to sebacic acid.

There were two main limitations in the previous decane induction process (by pouring/single bolus addition method) for achieving higher sebacic acid productivity (Fig. 1). First, an adjustment of the feeding rate of methyl decanoate (for decanoic acid adaptation) was required at an early period of methyl decanoate transformation. If this method was not followed, the fermentation would fail because of marked accumulation of decanoic acid and loss of the cell viability (Additional file 1: Figure S2). However, this solution was undesirable since it could decrease the sebacic acid productivity. Consecutively, higher feeding rate of methyl decanoate was an interesting proposition for enhancing the production of sebacic acid. Second, the accumulation of intermediates, especially decanoic acid, would pose extra risk for the stable production process because of its toxicity (Fig. 2). As reported earlier, decanoic acid was found to be cytotoxic for cells. Therefore, elimination of this compound during fermentation was a major objective in the biological production of sebacic acid.

Remarkably, substrate-limiting conditions during the decane induction period were effective enough for overcoming these limitations; it could especially eliminate the accumulation of decanoic acid as well and could remove the decanoic acid adaptation period in the early stage of methyl decanoate transformation (Fig. 5). Notably, this decane induction method provided a huge improvement in the biotransformation of methyl decanoate to sebacic acid (Table 1). Besides strain improvement, appropriate decane induction strategy was important to achieve a higher production of sebacic acid (Beardslee et al. 2014). Thus, the application of this method would have a significant effect in the large-scale production of sebacic acid.

**Table 1:** Research progress on the biotransformation of decane and methyl decanoate to sebacic acid

Microorganisms	Substrates	Titer (g/L)	Productivity (g/L · h)	References
*C. cloacae* 310	Decane	0.427	–^a^	Shiio and Uchio (1971)
Engineered *C. tropicalis*	Decane	0.94	–^a^	Chung et al. (2015), Beardslee et al. (2014)
Engineered *C. tropicalis*	Decane + methyl decanoate	± 13^b^	–^a^	Beardslee et al. (2014)
*C. tropicalis* mutant	Decane + methyl decanoate	27.0 ± 2.14^c^	0.50 ± 0.04	This study
*C. tropicalis* mutant	Decane + methyl decanoate	34.5 ± 1.10^d^	0.64 ± 0.02	This study

^a^ Not enough information

^b^ Decane induction by single bolus addition of 10 g/L decane (induction time: 6 h)

^c^ Decane induction by single bolus addition of 1% v/v decane (induction time: 10 h)

^d^ Decane induction by substrate limitation condition/continuous feeding (0.65 g/L/h decane for 10 h)

In addition, low solubility of decanoic acid in water could become a critical issue during this biotransformation process. In fermenter broth, methyl decanoate can be hydrolyzed to decanoic acid and methanol using lipases and esterase of *C. tropicalis* (Galán-Ladero et al. 2010). Practically, maintaining pH of 7.5 could ensure the solubility of decanoic acid. Because the pKa value of decanoic acid was lower than 7.5, it existed in a dissociated form in the fermenter broth and reacted with NaOH. The product of this reaction, sodium decanoate, was easily soluble in water. Notably, the main purpose of this pH control was for the solubility of sebacic acid instead of decanoic acid (Liu et al. 2004).

Additionally, the detail transport mechanism of 10-carbon chain of fatty acids was still debatable. Shorter carbon chains of fatty acids are transported by diffusion; however, longer carbon chains could be transported by either freely or by mediation of some protein (Hettema and Tabak 2000). Detailed study about these phenomena could be an interesting topic for further researches.

In the method described in the present study, the accumulation of 10-hydroxydecanoic acid was still detected in the fermenter broth even under decane limiting condition. Interestingly, this finding might be a proof that oxidation of 10-hydroxydecanoic acid is a bottleneck (the rate-limiting step) in the biological production of sebacic acid using *C. tropicalis*. Additionally, the accumulation of 10-hydroxydecanoic acid was always observed after a long time in biotransformation, usually after more than 60 h of the cultivation time (more than 40 h of biotransformation) even without increasing the methyl decanoate feeding rate (data not shown). Previously, evidence for a similar bottleneck in the production of dodecanedioic acid by *Yarrowia lipolytica* has been provided (Gatter et al. 2014; Werner and Zibek 2017). Further investigations are necessary to confirm these phenomena.

Because of the inhibition caused by decanoic acid (Figs. 3 and 4), elimination of this compound from the fermenter broth is essential for stabilizing the biotransformation process. Besides improving the oxidation of alkanes or fatty acids (Eschenfeldt et al. 2003; Funk et al. 2017), increasing the oxidation reaction rate of 10-hydroxydecanoic acid could be another important aim to further improve this biotransformation process. Various prospective solutions for increasing the specific activity of the enzymes involved in this reaction, such as overexpression of the relevant enzymes in *C. tropicalis*, cloning of alternative enzymes from other microorganisms, or protein engineering, would be required for overcoming this limitation. The enzymes of *C. tropicalis* involved in this reaction, especially fatty alcohol oxidase and fatty alcohol dehydrogenase, have been studied extensively (Kemp et al. 1988; Eirich et al. 2004; Lu et al. 2010). In this context, the overexpression of FAO1 gene of *Y. lipolytica* was reported to increase the productivity of dodecanedioic acid (Gatter et al. 2014).

As the conclusion, decane induction by continuous feeding would be essential for large scale biotransformation of methyl decanoate to sebacic acid. However, accumulation trend of 10-hydroxydecanoic acid was still observed even under decane limiting condition; this finding might be an early evidence that oxidation of 10-hydroxydecanoic acid is a bottleneck in the process when using *C. tropicalis.* To increase productivity of sebacic acid, study about these phenomena could be an important research area. Besides that, further investigation about transport phenomena of decanoic acid in the cell membrane also would be an interesting research topic in the future.

## Additional file

**Additional file 1.** Additional Figures S1, S2.

## Abbreviations

10-HDA: 10-hydroxydecanoic acid
DA: decanoic acid
DCAs: dicarboxylic acids
FAMEs: fatty acid methyl esters
FAO: fatty alcohol oxidase
PA: polyamide
SA: sebacic acid
YM: yeast mold
