# Supplementary material for: Effect of decanoic acid and 10-hydroxydecanoic acid on the biotransformation of methyl decanoate to sebacic acid
Source: AMB Express. 2018 May 5;8:75. doi: 10.1186/s13568-018-0605-4 (PMC5936482; doi:10.1186/s13568-018-0605-4)
Supplement: Supplementary file 1 — Additional file 1. Additional Figures S1, S2. [file 13568_2018_605_MOESM1_ESM.docx]

**Journal Name: AMB Express**

**Effect of decanoic acid and 10-hydroxydecanoic acid on the biotransformation of methyl decanoate to sebacic acid**

**Yohanes Eko Chandra Sugiharto^1,2^, Heeseok Lee^1,2^, Annur Dyah Fitriana^1,2^, Hyeokwon Lee^1^, Wooyoung Jeon^1^, Kyungmoon Park^3^, Jungoh Ahn^1,2^, Hongweon Lee^1,2^***

^1^Biotechnology Process Engineering Center, Korean Research Institute of Bioscience and Biotechnology (KRIBB), 30 Yeongudanji-ro, Cheongwon-gu, Cheongju-si, Chungcheongbuk-do 28116, Republic of Korea

^2^ Department of Bioprocess Engineering, KRIBB School of Biotechnology, Korea University of Science and Technology (UST). 217 Gajeong-ro, Yuseong-gu, Daejeon 34113, Republic of Korea

^3^Department of Biological and Chemical Engineering, Hongik University, 2639 Sejong-ro, Sejong-si 30016, Republic of Korea

* Corresponding author: **Hongweon Lee**

Telephone: (+82)-42-860-4740

Fax-number: (+82)-43-240-6609

E-mail: [hwlee@kribb.re.kr](mailto:hwlee@kribb.re.kr)

**Figure S1**

**Figure S1.** Effects of various substrates (
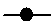
: decane;
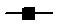
: potassium decanoate; and
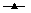
: methyl decanoate) on the production of sebacic acid in flasks with an evolved *C. tropicalis*. In cases of residual substrate, open triangles (
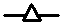
) indicate remaining fatty acid concentrations that was converted from methyl decanoate when methyl decanoate was used as substrate. Error bar represented standard deviation of two independent runs.

SA: sebacic acid; OD: optical density

**Figure S2**

**Figure S2.** Biotransformation of decane and methyl decanoate to sebacic acid using an evolved *C. tropicalis* . In this experiment, methyl decanoate was fed at 0.68 g/L/h after decane induction, and decanoic acid suddenly accumulated. Subsequently, fermentation failed because of foaming.

MD: methyl decanoate; DA: decanoic acid.

Symbol:
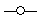
: optical density;
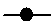
: sebacic acid concentration;
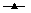
: decanoic acid concentration;
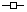
: decane concentration.
